# Climate Justice and the Duty of Restitution

**DOI:** 10.1515/mopp-2021-0071

**Published:** 2023-04-25

**Authors:** Santiago Truccone-Borgogno

**Affiliations:** Institute of Philosophy, University of Graz, Heinrichstraße 26/VI, Graz 8010, Austria

**Keywords:** beneficiary pays principle, burden sharing, excusable ignorance, historical emissions, legitimate expectations, restitution, unjust enrichment

## Abstract

Much of the climate justice discussion revolves around how the remaining carbon budget should be globally allocated. Some authors defend the unjust enrichment interpretation of the beneficiary pays principle (BPP). According to this principle, those states unjustly enriched from historical emissions should pay. I argue that if the BPP is to be constructed along the lines of the unjust enrichment doctrine, countervailing reasons that might be able to block the existence of a duty of restitution should be assessed. One might think that the duty to provide restitution no longer has moral weight if many benefits were already consumed, if the particular benefits obtained from historical emissions cannot be transferred from one country to another, or if present members of developed countries framed their life plans based upon the expectation of continued possession of those benefits. I show that none of these reasons negate the duty to provide restitution.

## 1 Introduction

The distinctive features of climate change require that we consider how climate mitigation and adaptation duties should be globally distributed. Given that present members of developing countries will suffer worse consequences of climate change than their counterparts in developed countries (Gough 2017, pp. 24ff.), and that they have obtained fewer benefits from historical emissions (Meyer 2013, pp. 612f., 2021, pp. 116ff.), it seems that present members of developed countries have a greater responsibility, and accordingly more significant burdens, in terms of mitigation and adaptation duties.1In this paper, I use the terms “developed countries” and “developing countries” since this is the terminology used in the Paris Agreement and most climate justice literature. However, I recognize that these terms are slightly controversial, and one might prefer to use instead terms such as “the Global North” and “the Global South”, or “formerly colonizing countries” and “formerly colonized countries”. However, present members of developed countries might object that it is unfair that they bear more significant burdens since many emissions were not released by them but by their predecessors (Caney 2006, p. 470), and that earlier emissions were released in a state of excused ignorance (Gosseries 2004, p. 359).2It is commonly accepted that these emissions refer to those released before 1990 when the International Panel on Climate Change (IPCC) published its first report. Further, currently living members of developed countries might claim that no present or future individual can be harmed (or benefited) by historical emissions since had those emissions not been released they would not be better off (or worse off) (Caney 2006, pp. 474f.). Hence, currently living members of developed countries might conclude that they should not bear a greater burden in remedying climate change.

In response to these objections, some authors have argued in favor of the beneficiary pays principle (BPP), according to which “the countries benefiting the most from greenhouse emitting activities in the past bear the greatest responsibility of climate justice” (Page 2008, p. 562; see also Barry and Kirby 2017; Duus-Otterström 2017).3This view seems to be grounded on Judith Thomson’s idea that innocent beneficiaries should pay some of the costs of dealing with some of the objectionable effects of the past acts since “they have profited from the wrongs the community did” (1973, p. 383). For a summary of different views grounded on this idea, see Pasternak (2017). In one interpretation, the BPP is constructed around the legal doctrine of unjust enrichment (Page 2012; Heyd 2017). In this view, “those states unjustly, but not wrongfully, enriched by activities that cause climate change should pay” (Page 2012, p. 308). This unjust enrichment principle justifies remedial duties without consideration of moral or even causal responsibility for the state of affairs to be redressed. The climatic unjust enrichment principle asserts that if the present generation of developed countries benefited from historical emissions at the expense of another party, they can be remedially responsible for redressing the current state of the climate crisis.

Part of the value of the unjust enrichment interpretation of the BPP is that even though it is a principle of corrective justice, it does not need to consider past emissions wrongful for assigning remedial responsibilities to developed countries.4Unlike the *polluters pays principle* (PPP) and the *wrongful enrichment* BPP. For a defense of these principles, see Neumayer (2000), and Butt (2014), respectively. Additionally, the unjust enrichment BPP, as Page (2012) calls it, diminishes some feasibility concerns. On the one hand, it entails that the distribution of climate duties be informed by historical considerations, as many developing countries claim.5See Singer (2016, pp. 37f.), Klinsky and Brankovic (2018, pp.13ff.). On the other hand, it also accounts for some of the preferences of many developed countries since it does not entail that they are morally responsible for their historical emissions.6See Anderson et al. (2017, pp. 450f.).

In this paper, I will not criticize the unjust enrichment interpretation of the BPP. I take for granted that present living members of developed countries have benefited from historical emissions, that those benefits were obtained at the expense of the currently living members of developing countries, and that those benefits are unjust.7This is according to the first three requirements for unjust enrichment: (1) one party has to have benefited; (2) the benefit has to be obtained at the expense of another party; (3) the benefit has to be unjust (Birks 1992, p. 41). However, I argue that if the BPP is to be constructed in accordance with the unjust enrichment doctrine, much more needs to be said in order for the developed countries to be bound by a duty of restitution. In unjust enrichment actions, the defendant can accept that she was unjustly enriched and still resist the duty to provide restitution. She can resist the claim by making use of defenses (Birks 1992, p. 41). Unlike *denials* that aim to show that there is no enrichment, or if there is that it is not unjust, *defenses* intend to show a further reason why the duty of restitution has been overridden or at least reduced in extent (Virgo 2015, p. 663). In the legal context, the distinction between unjust enrichment and duties of restitution is clear. As Stephen Smith asserts, “The law of unjust enrichment and the law of restitution are distinct fields. The law of unjust enrichment is a body of ‘primary law’ that identifies the situation in which an unjust enrichment arises. The law of restitution is a body of ‘secondary law’ explaining the existence and content of duties to make restitution” (Smith 2009, p. 202).

The discussion concerning the law of restitution tends to assess whether, despite the fact that some party unjustly benefited at the expense of another, there are further reasons that speak against the duty to restitute such a benefit or enrichment to another party. Defenders of the unjust enrichment BPP says almost nothing with respect to this second part.8Heyd briefly discusses as a defense that “industrial countries have been so accustomed to their technological achievements and material quality of live that a radical and sudden restitution would destroy their way of life and fabric” (Heyd 2017, p. 42). What Page calls chronological unfairness can also be understood as a defense (Page 2012, p. 317ff.). However, the non-identity problem (Parfit 1984, ch. 16) and what Page calls the disaggregation problem (2012, pp. 317ff.) would be best understood as denials (rather than defenses) since these objections intend to show that there is no enrichment, or that part of that enrichment is not unjust or it was not obtained at the expense of other parties. This paper is an attempt to complete their work by incorporating discussions about duties of restitution to the unjust enrichment interpretation of the BPP. Paying attention to how the legal literature has treated the unjust enrichment doctrine might shed light on some possible shortcomings in the approaches that practical philosophers have taken regarding the BPP.9Page recognized some years ago that the extensive legal literature on unjust enrichment “has yet to be applied systematically to climate change” (2012, p. 309). This is still not reflected in the climate justice discussion. In particular, I discuss some countervailing reasons that might be able to block the existence of a duty to provide restitution that arises from having unjustly benefited at the expense of another party. These reasons stem from changing circumstances. One might think that the duty to provide restitution no longer has moral weight if many benefits had already been consumed, if the particular benefits obtained from historical emissions cannot be transferred from one country to another, or if present members of developed countries framed their life plans based upon the expectation of continued possession of those benefits. I show that although these reasons are plausible candidates as defenses, none of them succeed in negating the duty to provide restitution. I argue that the unjust enrichment principle thus understood is able to justify that currently living members of developed countries have a duty to provide restitution towards presently living members of developing countries. I contend that, concerning mitigation burdens, the duty to provide restitution can be instantiated by allowing present members of developing countries to have a higher share of the benefits of the remaining global carbon budget (GCB) than the share they should have received had historical emissions not been taken into account.

## 2 Defenses Against the Duty of Restitution

In the climate justice discussion about distributing the remaining GCB, present members of developed countries can provide at least three reasons that might qualify as defenses. These potential defenses seek to show that even if they have unjustly benefited from historical emissions, they should not be bound by a duty of restitution or, at least, that such a duty should be reduced. They can assert that (a) it would be inequitable to restitute *all* the benefits obtained at the expense of the present members of developing countries since many of those benefits were consumed before present members of developed countries realized that those benefits were not theirs to keep. The second defense (b) asserts that the goods to be restituted under current circumstances are not the benefits from historical emissions but those of the remaining permissible emissions (the GCB). Since the benefits from the remaining permissible emissions are not the benefits from historical emissions but only something that can be considered their equivalent, currently living members of developed countries might claim that to engage in a remedial duty under such conditions would be unjustly harmful. The third defense (c) is that since present living members of developed countries have formed life plans relying on the benefits received at the expense of developing countries to engage in a duty of restitution would also be unjustifiably harmful.

The first defense (a) is, in Birks terminology, *enrichment-related*, while the other two (b) and (c) are *unjust-related* (Birks 1992, p. 126). Enrichment-related defenses tend to show that the enrichment or benefit suffered a detriment. In contrast, unjust-related defenses show that, even if there is not a detriment in enrichment, it is unjust to restitute the received benefit given how circumstances are now. These three defenses can be understood as *change of position* defenses of unjust enrichment actions.10This defense was recognized by Lord Goff in *Lipkin Gorman* as follow: “At present, I do not wish to state the principle any less broadly than this: that the defense is available to a person whose position has so changed that it would be inequitable in all the circumstances to require him to make restitution, or alternatively to make restitution in full” (Bant 2009, p. 125). One might think that the *bona fide purchase* defense also applies. One can assert that present members of developed countries should be protected because they entered into possession of the benefits of historical emissions in good faith. However, this defense requires something that is missing in the climate justice debate. Even if currently living members of developed countries came into possession of the benefits in good faith, they did not *purchase* but merely *received* them from their predecessors. The justification of this *bona fide purchase* defense lies in the fact that “if purchasers are at risk of unexpected liabilities on account of the assets they buy, they’ll be discouraged from entering in such transactions” (Webb 2016, p. 230). In the climate justice debate, however, there is no contract or purchase between current or past members of developing and industrialized countries to protect. One way of translating the *change of position* defense into the climate justice debate is by asserting that the defense entails that due to changing circumstances the backward-looking duty to provide restitution is superseded by forward-looking distributive justice-based reasons.11See Waldron (1992).

The general rationale of the *change of position* defense*,* as Charlie Webb explains, is that “the claimant interests cannot be secured in full without doing harm to the defendant” (Webb 2016, p. 226). Suppose it is accurate to claim that many benefits from past emission-generating activities have already been consumed by pre-1990 activities of blamelessly ignorant present members of developed countries, as in (a). In this case, requiring full restitution of all their benefits that stem from historical emissions obtained at the expense of developing countries will harm currently living members of developed countries. It is also harmful that they have to give up something different than the received good, as in (b), and have to provide full restitution which prevents them from enjoying those remaining benefits upon which they have framed and organized their life plans, as in (c). Hence, for each of these situations, as in the legal context in which the defense of change of position is pleaded, “the question is whether restitution should nonetheless be ordered and this harm is then done to the defendant or, alternatively, whether restitution should be denied, leaving the claimant instead to suffer the loss” (Webb 2016, p. 226).

## 3 The Benefits Are No Longer Here

Present members of developed countries have benefited from historical emissions in several ways. Current available infrastructure is probably the clearest example of benefits enjoyed today owed to past emission generating activities as many of them were built before currently living individuals were even conceived. Among other things, this infrastructure includes railroads, streets, health and educational systems (Meyer 2013, pp. 606ff.), public parks, pipelines, sewers, electrical and gas networks. Furthermore, the benefits from past emission generating activities enjoyed by currently living people consist not only in being wealthier but also in being less vulnerable to the adverse effects of climate change (Meyer and Roser 2010, p. 231). However, some benefits obtained at the expense of developing countries have not endured but were consumed before the present members of developed counties knew or were required to know that they were benefiting from those emissions that contributed to climate change. The first version of the change of position defense focuses on these already consumed benefits and aims to reduce the duty of restitution. According to this version of the defense (a), it would be inequitable that present members of developed countries have to restitute *all* the benefits received at the expense of currently living members of developing countries since many of these benefits were consumed by themselves before they knew that the benefits did not belong to them.12To be sure, this version of the defense does not intend to discount the extent of the duty of restitution concerning those benefits presently enjoyed by present members of developed countries but only those already consumed by them before the problem of climate change was widely known.

This version of the defense aims to reduce the duty of restitution because there was a detrimental change of position.13The detrimental change of position of present members of developed countries can be understood as a de-enrichment and as something irreversible since the benefits already consumed cannot be recovered. Therefore, supporters of both the *de-enrichment* and those of the *irreversibility* approach concerning how to understand detrimental change of position should have no complaint that this condition obtains here. On this, see Bant (2009, pp. 126ff.). According to the unjust enrichment legal doctrine, however, the fact that the received benefit was reduced is not enough for the defense to succeed. Typically, the defense succeeds if fulfilling the duty of restitution entails that the defendant is left worse off than he would have been had he not received the benefits. However, even if the defendant would be left in that position, the defense fails in two particular circumstances. First, if the enriched party knows that the received benefits do not belong to him. Second, when the defendant would have consumed those benefits anyway, typically because there is no causal connection between the receipt of the benefits and the expenditure.14Thanks to an anonymous reviewer for pressing on this point.

Given that it is reasonable to sustain that currently living members of developed countries had no knowledge before 1990 that those benefits were theirs to keep, we should analyze whether they would have consumed those benefits anyway. The defense can succeed if there is a “causative link between the receipt of the benefit by the defendant and his or her change of position, so that, but for the receipt of the benefit, the defendant’s position would not have changed” (Virgo 2015, p. 682). This condition requires a causative link between the inflow and the outflow in the sense that the benefited party incurred some expenditure only because he or she received the benefit. In absence of such causal connection, the defense fails.

In a classic example, the defendant received, for instance, £1000 and, because of this, he spent £200 on a party (Birks 1992, p. 135). Had he not received £1000, he would not have spent £200 on a party. In this situation, if the defendant had no reason to believe that the benefit was unjustified at the moment of the expenditure, he would only have to restitute £800. For instance, in the 1991 English precedent *Lipkin Gorman (a firm) v Karpnale Ltd.,*15Lipkin Gorman v. Karpnale [1988] UKHL12 (June 6th, 1991): [1991]3WLR10, [1988] UKHL12, [1991]2AC 548. the defendant put forward that its position changed when it paid winnings to the thief. In this case, a firm’s partner used money from the company’s bank account to gamble at a club in London (property of Karpnale Ltd.). The company was unable to sue the partner since he was in prison and without money. Instead, the company sued the club and succeeded in recovering the money (Birks 2004, p. 514). However, the club was able to prove that “but for [it]’s belief that the thief was entitled to gamble with the money, it would not have paid the winnings to the thief” (Virgo 2015, p. 687). The defendant was able to show a causative link between its belief that the thief was entitled to gamble and the expenditure. In this case, although the defendant was unjustly enriched at the plaintiff’s expense, it successfully showed that the enrichment suffered a detriment that ought to be taken into account in defining the extent of the duty of restitution.

Currently living members of developed countries might try to provide a similar argument concerning the benefits they consumed before 1990. They might try to show a causative link between the receipt of the benefit and their detrimental change of position. They might claim that, *but for* their belief that they were entitled to use those benefits, they would not have consumed them as they did. Thus, those benefits consumed before 1990 should not be considered in determining the duty of restitution. In other words, they consumed those benefits *only because* they did not know that they were obtained at the expense of currently living members of developing countries. Had they known this, they would not have consumed the benefits. Instead, they would have restituted them to currently living members of developing countries.

In our case, this defense can succeed if it can be shown that knowing the status of those benefits would have made a difference in how currently living members of developed countries behaved. This would be so because in this case developed countries (defendant) would be able to show a causal connection between the receipt of the benefits and their consumption. If it is certain, or at least, highly probable, that such knowledge would have made no difference in how they behaved, then we have no reason to believe that this defense can succeed. Thus, we need to assess whether having such knowledge would have made a difference in how currently living members of developed countries behaved.

Discussion in the literature is scant about whether currently living members of developed countries would have consumed benefits that stem from historical emissions if they had known that those benefits were obtained at the expense of currently living members of developing countries. Still, it is possible to find analyses concerning a somehow analogous topic, namely, whether developed countries would have emitted GHGs as they did if they had known of the harmful effects of climate change.16For instance, Zellentin (2014), and Butt (2017). The discussion on the latter issue, I think, sheds light on the former.

Concerning historical emissions and after recognizing that people cannot be morally responsible for harmful effects of the emissions released in a state of excused ignorance, Daniel Butt argues “that more advanced scientific knowledge would have made no difference to the actions of the emitters or to those responsible for regulating them: even if they had known of the likely consequences of their actions, they would have carried on regardless” (2017, p. 66). Butt’s general thesis is that if an agent acted in a state of ignorance concerning a particular effect of his act, and knowledge of that effect would have likely made no difference in how the agent behaved, such ignorance cannot block the attribution of remedial responsibility for the outcome actually produced (Butt 2017, p. 66).

Butt’s argument faces two challenges: an epistemic one and a normative one. The epistemic challenge rests on identifying whether people would have acted as they did if they had known the relevant information. The normative challenge questions whether such a counterfactual condition is relevant for establishing the morally faulty character of the historical past emitters and, accordingly, for assigning remedial responsibility (Butt 2017, p. 67).

Although I am sympathetic to Butt’s view, for the purposes of this paper, it is not necessary to accept that someone can be held remedially responsible if knowledge of the effects of some action would not have made a difference in how the agent behaved. For the change of position defense to succeed, the only point in need of proof is whether *but for* the belief of present members of developed countries that they were entitled to use those benefits, they would not have consumed them as they did. I am not arguing that a person can be held remedially responsible for a certain state of affairs caused by her if she would have behaved as she did had she known about the harmful effects of her action. This claim is much stronger than the one I need to show here. For my purposes, I only need to show that knowledge about the status of the benefits would have made no or a slight difference in how currently living members of developed countries behaved. I face only the epistemic challenge. This is because the change of position defense proceeds on the assumption that the *but for* condition is central for blocking the restitutionary duty. Thus, if I can show that the condition is not fulfilled, this is enough to reject the defense.

As I argue below, I believe that the reasons Butt provides for showing that knowledge about the harmful effects of historical emissions made no difference in how developed countries behaved also speak in favor of considering that the knowledge of the fact that the benefits of historical emissions were obtained at the expense of currently living members of developing countries would also have made no difference in how currently living members of developed countries consumed them. First, Butt highlights that developed countries are not particularly empathetic in their dealings with respect to the harms suffered by developing countries. It is commonly understood that knowing the sufferings and harms developing countries will undergo does not make a significant difference in how developed countries behave. As Butt asserts, they tend to privilege national interests, despite the harms other countries might suffer as a consequence of their actions (2017, p. 67). We might think not only about how developed countries behaved historically concerning emissions of GHGs, as Butt does, or about how developed states have strong preferences for increasing national security budgets by cutting their aid to relieve extreme global poverty, even in times of peace,17See García-Gibson (2017). but also about how they are now dealing with the COVID-19 pandemic and global vaccine distribution. As of February 2021, more than half of all COVID-19 vaccine doses were purchased by developed countries. These states, however, only represent 16% of the world population. Further, only ten countries had administrated more than three-quarters of the total vaccines available at that time.18See Yamei (2021). As of today, although 64% of the world’s population has received at least one vaccine dose, this number decreases to 14.4% when we only consider people living in low-income developing countries.19See Our World in Data. Retrieved on March 23, 2022, from https://ourworldindata.org/covid-vaccinations. For an illuminating analysis of how little progress has been made concerning global vaccine distribution and how developed states strongly prioritize national self-interests in this regard, see Holzer et al. (2022). These facts do not seem to speak in favor of developed countries being particularly empathetic toward developing countries. Thus, they do not seem to favor the view upon which developed countries would have behaved differently had they known that they were consuming benefits obtained at the expense of developing countries. On the contrary, it seems such knowledge makes no difference at all.20The fact that developing countries’ harms make no or a little difference in the behavior of developed countries in their dealings with them is strengthened if Thomas Pogge’s point is accepted about how developed countries play a prominent part in those harms suffered by developing countries. In his view, the global institutional order is prominently shaped and upheld by developed countries (Pogge 2004, p. 265). In Pogge’s view this order is not only better for developed and worse for developing countries, but it also has an important role in the maintenance and promotion of those domestic factors that prevent developing countries from becoming developed (Pogge 2004, pp. 272ff.). In this view, the problem concerning developed countries’ behaviors is not so much that they are indifferent to developing countries’ harms, but rather that developed countries are involved in causing harm to developing countries.

The second consideration Butt gives for showing that awareness about the harmful effects of historical emissions would have made no difference in how developed countries behaved is that “the development of knowledge about the effects of GHG emissions has not, in fact, served to check emissions in any kind of meaningful way” (Butt 2017, p. 68). A similar point seems to apply to the consumption of the benefits that stem from historical emissions obtained at the expense of present members of developing countries. Even after developed countries were aware, or at least, after they should have been aware, that the benefits from historical emissions were obtained at the expense of developing countries, they continued emitting as if benefits would not have been obtained at the expense of other countries. As of today, GHG emissions have not been reduced at acceptable rates by most developed countries.21As highlighted by Williges et al. “applying a production-based accounting approach of the 36 OECD countries, 10 had increasing emissions from 1995 to 2015, while using a consumption-based approach 14 nations had such” (Williges et al. 2022, p. 3). Furthermore, most countries’ targets and actions are insufficient (many highly or critically so) to align with the Paris Agreement temperature limit. See https://climateactiontracker.org/countries/ [Accessed March 15, 2022]. Given this, it is hardly true that present members of developed countries would not have consumed many benefits from historical emissions had they believed (before 1990) that they were not entitled to use those benefits. After knowing that many benefits were obtained at the expense of developing countries, not only did developed countries not return them, but they continue to obtain even more benefits at the expense of the present and future members of developing countries.22The current behavior of the present members of developed countries concerning those benefits speaks in favor of increasing rather than decreasing the duty of restitution towards developing countries. The more benefits obtained at the expense of developing countries are consumed by the developed ones, the fewer benefits from the rest of permissible emissions remain for being used by the former.

If this reasoning is accepted, this version of the change of position defense does not succeed. This is because it is highly likely that knowing that many benefits from historical emissions were obtained at the expense of the present members of developing countries would have made no difference in how the present members of developed countries behaved. In a relevant sense, there is no causative link between the inflow and the outflow. It is not the case that *but for* their belief that they were entitled to use those benefits, currently living members of developed countries would not have consumed those benefits as they did. Therefore, those benefits consumed by presently living members of developed countries before they were aware that those benefits did not belong to them should be included in the duty of restitution.23Therefore, this view differs from both Meyer’s distributive justice BPP and Heyd’s unjust enrichment BPP. Meyer seems to accept that the benefits consumed by currently living people when they were blamelessly ignorant about the harmful consequences of climate change “should be considered irrelevant for the distribution of the remaining permissible global carbon budget” (2021, p. 120). In Heyd’s view, “what decides the extent of our duty now is the size of our actual benefit from the original act, however (unintentionally but objectively) harmful it turned out to be” (2017, p. 40).

## 4 The Benefits Are Not the Same

The second defense (b) also recognizes that presently living members of developed countries unjustly benefited at the expense of present members of developing countries. However, it objects that the goods to be restituted under current circumstances are the benefits of the remaining permissible emissions (the GCB). Since the benefits from the remaining permissible emissions are not the benefits from historical emissions but only something that can be considered their equivalent, it has to be assessed whether currently living members of developing countries have a valid claim to receive that equivalent good due to the unjust enrichment of the former. What reason, if any, can justify this shift?

This shift requires justifying why the duty of restitution can be instantiated by providing a substitute or equivalent good to the specific benefit that gave rise to it. In our context, the problem is that to oblige present members of developed countries to instantiate the duty of restitution not by returning the good obtained at the expense of developing countries but by providing an equivalent good seems to imply that the former is unjustly harmed. The issue is that to allow this kind of shift could make the practical implication of the unjust enrichment principle overdemanding. As Avia Pasternak explained, most present people find themselves in a situation in which almost all the benefits they enjoy can be correlated with some past injustice (Pasternak 2017, p. 419). Thus, some kind of limit with respect to the scope of the duty of restitution is needed.24See Goodin (2013, pp. 485ff.), Pasternak (2017, pp. 418ff.).

In standard cases of unjust enrichment, the object of the duty of restitution is the very same good or asset that the enriched party used without the authorization of the harmed one. If someone transfers £150 of mine to you without my authorization (or if I transferred the amount by mistake), the duty of restitution usually consists of you returning the £150 to me. However, the duty of restitution concerning the remaining permissible emissions has to be different. Past members of developed countries have already emitted GHGs at the expense of currently living members of developing countries. These emissions benefited not only themselves but also their succeeding generations. The problem is that neither the benefits already consumed nor those remaining in possession of currently living members of industrialized countries can be returned, but only something different.

It is true that but for the fact that past members of developed countries emitted a high amount of GHGs, present members of developing countries could have benefited more from their own emissions. However, it is also true that the historical emissions of past members of developed countries contributed uniquely to the well-being and way of life of the present members of those developed countries. Further, many benefits cannot be physically transferred from one country to another, and the benefits enjoyed by currently living members of developed countries contain the effort of their preceding members when they emitted GHGs.25We can say, borrowing Locke’s expression for issues of acquisition of resources, that when past people *mixed their labor* (1970, p. 288 [27:6]) in emitting GHGs, the benefits currently living people enjoy also contain the efforts of their preceding generations. In this way, the benefits that present members of developed countries enjoy are unique and arguably not subject to restitution. Given these facts, it makes no sense to demand the restitution of the specific benefits obtained from those past emission-generating activities.

In the law of restitution two cases are often distinguished when the plaintiff demands the restitution of something different than the very thing that constituted the “unjust enrichment.” The first and stronger case in favor of restitution occurs when the plaintiff demands to receive something that is derived from the enrichment. Consider again the example in which someone transfers £150 of mine to you without my authorization. In such a case, you may have already spent that money on buying, for instance, a bicycle. However, this fact does not, by itself, exempt you from fulfilling the duty of restitution. In this case, if the unjust enrichment action succeeds, you are obligated to give back either the £150 or the bicycle, since you spent the £150 mistakenly transferred to you to buy it.26Something like this seems to be what Goodin has in mind when he limits the scope of the duty of restitution to the fruits of the injustice. He asserts that not all the things causally connected with the injustice are the appropriate target of the duty of restitution. In his view, that duty is restricted either to the object that has passed from one agent to the other, or its substitutes, that is the “new things that have taken place of the old” (2013, p. 486). The second case occurs when the plaintiff demands something that it is not directly derived from the initial enrichment.27Thanks to an anonymous reviewer for highlighting this distinction. These cases are more difficult. As Webb asserts for legal contexts, in this kind of situation, “there can, therefore, be no return or reallocation of these benefits, only offsetting, only the imposition of some measure which worsens the defendant’s position, setting back his goals and interests, in equal measure to the advancement his use of the asset provided” (Webb 2016, p. 187).

The restitution sought in the climate scenario is more similar to the second kind of case. For climate justice-based duties of restitution, present members of developing countries might demand that, if the specific benefits obtained at the expense of developing countries or something causally derived from them cannot be returned (as seems to be the case), then present members of developed countries should give up something equivalent to the value of the good they have unjustly obtained at the expense of currently living members of developing countries. In particular, the suggestion is that the duty to make restitution has to be instantiated by allowing currently living members of developing countries to have a larger share of the GCB than the share they would have received had the historical emissions not been taken into account. They should be allowed to benefit from an “extra” share of the remaining GCB.

The fact that past emissions of developed countries had the effect of reducing the available options of the present members of developing countries provides a way of justifying that the object of restitution consists of allowing developing countries to obtain a larger share of the GCB. Concerning the duties of climate mitigation, as Page asserts, past emissions of developed countries caused currently living members of developing countries to face the tragic dilemma of: (a) either fulfilling their mitigation duties with the cost of not being able to develop their economy, and in some cases to reduce severe poverty, or (b) using cheap but polluting sources of energy so that they can industrialize quickly but at the cost of not being able to fulfill their mitigation duties (Page 2012, p. 315).28Concerning adaptation duties, something similar occurs. Suppose these developing countries invest their resources in improving their economic conditions so that poverty is reduced. In that situation, it might be that they cannot use those resources to build infrastructure to counter the adverse effects of climate change and vice versa. This does not deny that there could be synergies between building infrastructure to improve the quality of life or in favor of the industrialization of a given country and that those assets could also serve as a means of adaptation to some of the adverse effects of climate change. However, this need not be the case. The fact that historical emissions of developed countries benefit their present members makes it reasonable to ask them to take care of the harms and losses present members of developing countries have to face due to those historical emissions. In cases of unjust enrichment, although one party is harmed, there is no need, and it is often not the case that such harm has occurred as a consequence of wrongdoing committed by another party. Still, there is no objection in claiming that those who have unjustly benefited at the expense of another should remediate the harm suffered by another party, even if in doing so they can only provide a different good from the one the benefited party has obtained.

Consider the following case of mistaken payment:

Here [when I mistakenly pay you money], requiring you to make good my loss doesn’t set back your interest. By using the £100 I paid you to pay your phone bill, you were able to save £100 of your own. If you now have to pay me £100 you are left in the same position you would have been in had I not made the payment to you, no better but no worse off (Webb 2016, p. 195).

Similarly, when we consider the unjust enrichment of present members of developed countries at the expense of developing countries, we can claim that requiring the former to make good the loss of the latter does not set back the former’s interests even if in doing so they have to provide not the specific benefits they obtained but an equivalent good. Imagine a developed country that was able to expand its railroad system owing to those historical emissions released at the expense of currently living members of developing countries. By releasing those emissions that would have belonged to currently living members of developing countries, they saved their own share of emissions for obtaining further benefits, for instance, an advanced system of public education or a robust health system. If currently living members of developed countries have to pay to currently living members of developing countries employing some equivalent good of that already used, they would, at most, be left in the same or equivalent position as they would have been had they themselves and their ancestors not emitted GHG at the expense of currently living members of developing countries.

As in the mistaken transfer example, we are not shifting losses from one party to another but only ensuring that such loss is not suffered. The railroad system of this highly developed country indeed came from how its past members caused emissions. Moreover, it is also true, as Webb argues, that “the simple fact that you now have items of wealth at your disposal as a result of your unauthorized use of my assets gives me no interest in, and so no claim to, those items” (Webb 2016, pp. 195f.). Therefore, there is no doubt that railroads and other benefits stemming from historical emissions belong to currently living members of this highly developed country. However, this is not the issue. In this understanding of the duty of restitution, it is not a question about whether or not those benefits belong to currently living members of developed countries. The issue is whether present members of this developed country can justifiably resist the claim of present members of developing countries that the former should remedy the current situation, on the basis that their interests are unfairly set back when they give developing countries a good equivalent to the benefits obtained at their expense. In these cases, what is relevant is that this equivalent good, in the climate case a larger share of the benefits of the remaining permissible emissions, is something that the present members of developed countries would not have but for the fact that their preceding members emitted GHGs at the expense of present members of developing countries.

To the extent of this equivalent good that developed countries have saved at the expense of developing countries, as Webb asserts for cases of money already spent, “requiring you [the enriched party] to make good of this harm leaves you worse off only by offsetting an advantage which ought not to have come your way, an advantage you made through (unknowingly) doing me [the other party] harm” (Webb 2016, p. 196). Requiring present members of developed countries to make good on the harm suffered by present members of developing countries leaves the former worse off only by offsetting an advantage they should not have had, which they obtained through (wrongless) harming of the present members of developing countries. Therefore, to oblige present members of developed countries to instantiate the duty of restitution by employing an equivalent good to that obtained at the expense of developing countries does not imply that the former is unjustly harmed. Hence, this instance of the change of position defense has to be rejected.

## 5 Benefits, Life Plans, and Status Quo Expectations

The third possible defense (c) arises as follows. Present members of developed countries benefited from the GHG emissions of their predecessors. In light of the unjust enrichment principle, those benefits were unjustly obtained at the expense of the present members of developing countries. Therefore, there are strong reasons for the present members of developed countries to be bound by a duty of restitution based on how those benefits were obtained. Since the specific benefits obtained from historical emissions of GHG cannot be restituted, the duty of restitution has to be instantiated by providing to the present members of developing countries an equivalent good. In particular, such a duty of restitution has to be instantiated by giving present members of developing countries a higher share of the remaining GCB than the share they should have received had the present members of developed countries not benefited at their expense. However, there is a limit to the upper level of emissions the present generation of both developed and developing countries can release to not wrongfully harm future people (Meyer and Roser 2006, p. 226). Given this upper limit, fulfilling the duty of restitution implies that the present members of developed countries have to reduce their GHG emissions substantially (Truccone-Borgogno 2022, pp. 369ff.). The problem is that such a reduction will frustrate some of their historically formed expectations.29If the reduction is too radical, it can also be unfeasible. In the climate literature it is sometimes considered that a feasible reduction rate of global reduction of GHG emissions consists of halving them each decade. This is the so-called “carbon-law” (Rockström et al. 2017). I assume that the domestic reduction rate is compatible with this carbon law. As Meyer asserts, “people living in countries with a high level of per-capita emissions typically have a wide range of expectations that are likely to be frustrated if climate change is dealt with appropriately” (2021, p. 125). Based on these considerations, defense (c) asserts that present members of developed countries formed life plans based upon the expectation of continuing, at least temporarily, to emit high amounts of GHG. Since engaging in the reduction required by the duty of restitution is incompatible with the fulfillment of those expectations, it would be unjust to require them to fulfill such a restitutionary duty.

In unjust enrichment actions, the defense based on the frustration of the expectations of the defendant is typically exemplified with the case in which the plaintiff mistakenly transfers money to the defendant who, as a consequence, believes that she is entitled to use and spend it. Here, even if the defendant is enriched at the plaintiff’s expense, the change of position defense usually succeeds since the former was led to believe by the latter that the benefits obtained are her own. Since the defendant has no responsibility in the formation of the expectation that she can use the money as if it were her own, she makes plans based on the assumption that the money is hers to keep. Charlie Webb asserts that “the harm [the defendant] would suffer in these cases comes from [her] assumption that the money [she] receives from me was [hers] to keep, that [she] could go ahead and plan and spend on that basis” (Webb 2016, pp. 221f.). In this situation, if this person has to restitute the received good, then she is unjustly harmed because her expectation based on the possession and ownership of the received good is frustrated.

Something similar seems to occur when we analyze the duty of restitution that obliges present members of developed countries to reduce their emissions substantially. Like the person who received money mistakenly transferred from the claimant, present members of developed countries have no responsibility in the formation of the expectation of being able to carry on emitting higher levels of GHGs. Since they have formed life plans based on these expectations, like the person who received a mistaken transfer, to oblige them to reduce their level of emissions substantially seems to imply that they would be unjustly harmed.30Based on this reasoning, these developed countries might try to justify a scheme of distribution of the GCB in accordance with the contraction and convergence view. This view “derives an allocation of the remaining budget based on today’s unequal level of emissions (thus favoring mainly wealthy, high emitting countries) and population shares, with convergence to an equal per capita distribution by the end of the budget period” (Williges et al. 2022, pp. 1f.).

To be sure, although the frustration of expectations is often harmful, only those expectations that are legitimate are normatively relevant.31As an anonymous reviewer reminded me, in the law of restitution merely defeating expectations isn’t enough for making a case for the change of position defense. In their view, we also should be able to identify some setback to the benefited party’s interests to justify denying their unjust enrichment liability. Relying on the notion of legitimate expectations intends to explain exactly this point. It is not the fact that the expectations of members of developed countries might be frustrated but the fact that their *legitimate* expectations would be undermined that raises a prima facie change of position defense. As Meyer asserts “one of the implications of a legitimate expectation is that its bearer has a valid normative claim that the harm that can result from the frustration of the expectation is taken into account when decisions and actions are taken that can lead to its frustration” (Meyer 2021, p. 125). Expectations are legitimate if they have epistemic and justice-based validity. Epistemic validity occurs when there are good reasons for thinking that some expectation will be fulfilled in the future, while justice-based validity occurs when an expectation has the proper connection to justice (Meyer and Sanklecha 2014).32For a detailed explanation of how expectations might have epistemic and justice-based validity, see Meyer and Sanklecha (2014, p. 383ff.) and Meyer and Truccone-Borgogno (2022). For our discussion, it is useful to look at the epistemic validity of present members of developed countries’ expectations concerning their level of emissions. In this view, “epistemic validity occurs either because some past behavior or circumstance gives reasons to believe that the future will be akin to the past in virtue of which the expectation is formed, or because those who have the power to frustrate another’s legitimate expectation announce that this will not be the case” (Meyer and Truccone-Borgogno 2022, p. 4). Recall the mistaken transfer case. Here the claimant plays a role and contributes to the defendant’s belief that she is entitled to use the received money. In this case, the claimant’s behavior is the source of the epistemic validity of the defendant’s expectation. This fact explains why the defense is stronger in this case. In unjust enrichment actions, when the claimant creates the expectation that the defendant can use and rely on the assets transferred, the former is responsible for that situation. In these cases, the defense of change of position succeeds (Webb 2016, pp. 226ff.).

However, the case for the defense is not as strong and can be rejected when the plaintiff plays no role in creating the expectation that the defendant can rely on the benefits obtained at his expense (Webb 2016, p. 226f.). In the climate debate, present members of developing countries played no role in the formation of currently living members of developed countries’ expectations to continue emitting high amounts of GHG. Thus, one might think that the defense must be rejected. This response would be too quick. Although present members of developing countries did not contribute to the formation of those expectations of currently living members of developed countries, which might have to be frustrated in order for their states to fulfill their climate duties of restitution, currently living members of developed countries have no responsibility either.33Thanks to an anonymous reviewer for pushing me to address this issue. Therefore, it seems that some of the present members of developed countries’ expectations concerning their future levels of emissions of GHG deserve some kind of protection. However, such a protection does not play a role in the international discussion about whether developed countries might resist the unjust enrichment-based duty of restitution towards developing countries. Instead, the protection has its stronger form against their own governments when these developed states design policies of domestic distribution of the remaining permissible emissions. This is because, as Meyer asserts, “highly industrialized states have played a constitutive role in shaping [the status quo] expectations [of their residents] and continue to do so” (2021, p. 130; also, Williges et al. 2022, p. 6). It is for this reason that in discussions of international justice, developed countries cannot rely on this version of the change of position defense for resisting the claim for restitution by developing countries. As explained above, developed countries (defendant) cannot claim that they bear no responsibility in the formation of the expectations of their members that now have to be frustrated in order for these countries to fulfill their international climate duties of restitution towards developing countries (plaintiff). Since developed countries are not innocent but instead responsible for forming and shaping the expectations of their members, they cannot rely on this version of the change of position defense.34For similar reasoning, the “Estoppel” defense has to be rejected. This defense protects the recipient of the benefit when she is led to believe that the benefit was validly received. The success of the defense barred the claim of the plaintiff completely (Virgo 2015, p. 666). For the climate justice debate, one might think that since the current generation of developed countries were led to believe that they can enjoy the benefits from past emission generating activities, the claim for restitution should be rejected. However, this defense does not succeed because it requires that the plaintiff be responsible for making the defendant believe that they could enjoy the received benefits (Virgo 2015, p. 667), as it occurs in cases of mistaken payments. In the climate justice debate, however, currently living members of developing countries never did anything that could have led currently living members of developed countries to believe that they are entitled to enjoy the benefits from past emission generating activities. This is because those benefits have been conferred, not by present members of developing countries, but by preceding members of the developed countries.

## 6 Conclusion

In this paper, I started from the assumption that having unjustly benefited at the expense of another generates strong reasons in favor of a remedial duty of restitution. In particular, I argued in favor of allowing developing countries to have a higher share of the benefits of the remaining GCB than the share they should receive had historical emissions not been taken into account. I assessed three possible defenses against such a duty and argued that all of them fail. The first defense asserts that since many of those benefits had been consumed before present members of developed countries knew that those benefits did not belong to them, having to restitute them would cause unjust harm. As I argued, in the unjust enrichment doctrine, for a detrimental change of position to serve as a defense it needs a causative link between the receipt of the benefit and the change of position. Since it is highly likely that present members of developed countries would have behaved as they did even if they had known that those benefits did not belong to them, I argued that the *but for* condition is not met. It is not the case that, *but for* present members of developed countries’ belief that they were entitled to use those benefits, they would not have consumed those benefits as they did.

The second defense objects that the goods to be distributed under current circumstances are the benefits of the remaining permissible emissions and not those received from historical emissions. Thus, to instantiate the duty of restitution in this way would be unjustly harmful. Against this objection, I argued that from the perspective of unjust enrichment actions, it is not the question whether or not the specific benefits that stem from historical emissions belong to currently living members of developed countries. Instead, it is questioned whether present members of the developed countries can resist the claim of present members of the developing ones that the former should remedy the current situation. As I argued, what is relevant is that the equivalent good that is claimed is something that developed countries would not have but for their historical emissions. Thus, there is no objection that the duty of restitution will be instantiated by allowing present members of developing countries to have a higher share of the benefits of the remaining GCB than the share they would have received had historical emissions not been taken into account.

The third defense is that it would be unjust to require present members of developed countries to fulfill the restitutionary duty since they have formed life plans based upon the expectation of continuing to emit high amounts of GHGs and that to engage in the reduction required by the duty of restitution is incompatible with the fulfillment of those expectations. Against this defense, I argued that present members of developing countries did not contribute to the formation of currently living members of developed countries’ expectations regarding the continuation of high levels of GHG emissions. Instead, those expectations were formed by developed countries themselves. For this reason, these countries cannot claim that they have no responsibility in the formation of the expectations of their members. Therefore, the change of position defense based on the status quo expectations of the members of developed countries has to be rejected. Currently living members of developed countries have a duty to allow present members of developing countries to have a higher per-capita share of the benefits of the remaining GCB than the share they should have received had historical emissions not been taken into account.
